# Psychological well-being and mortality: longitudinal findings from Lithuanian middle-aged and older adults study

**DOI:** 10.1007/s00127-019-01657-2

**Published:** 2019-01-09

**Authors:** Abdonas Tamosiunas, Laura Sapranaviciute-Zabazlajeva, Dalia Luksiene, Dalia Virviciute, Anne Peasey

**Affiliations:** 1 Institute of Cardiology, Medical Academy, Lithuanian University of Health Sciences, Kaunas, Lithuania; 2 Health Psychology Department, Lithuanian University of Health Sciences, Tilzes str. 18, Kaunas, Lithuania; 3 Institute of Epidemiology and Health Care, University College London, London, UK

**Keywords:** Psychological well-being, Mortality

## Abstract

**Background:**

The study aimed to examine whether after confounding by possible socio-demographic and other risk factors, psychological well-being is independently associated with reduced all-cause and cardiovascular mortality.

**Methods:**

Initial data were collected within the framework of the international project HAPIEE in 2006–2008. A random sample of 7115 individuals aged 45–72 years was screened. Deaths were evaluated by death register of Kaunas city (Lithuania) in a follow-up study till 2016. Psychological well-being was evaluated by a CASP-12 questionnaire. Socio-demographic, lifestyle, biologic factors and depressive symptoms were evaluated.

**Results:**

Age-adjusted survival curves revealed that psychological well-being predicts longevity in men and women (*p* < 0.001). After adjustment for many possible confounders psychological well-being was independently associated with all-cause mortality in men (HR 0.77; 95% CI 0.62–0.94) and women (HR 0.73; 95% CI 0.56–0.96). However, psychological well-being association with cardiovascular mortality attained statistical significance only in the women’s group (HR 0.53; 95% CI 0.33–0.87), but not in men (HR 0.98; 95% CI 0.72–1.33).

**Conclusions:**

Psychological well-being is an important predictor of longevity, controlling well-recognized risk factors such as age, education, cardiovascular diseases, social status, marital status, lifestyle and biological factors and depressive symptoms. Positive psychological well-being should be taken into account when screening older people to prevent negative health outcomes.

## Background

It has been known that cognitive and personality traits of a person are associated with health outcomes like mortality [1, 2]. Moreover, there is growing evidence that dynamic psychological aspects of a person, like emotions, might be linked with mortality too [3, 4]. Some studies suggest psychological well-being’s (PWB) effect on long-term survival [5–8], especially its protective role from CVD mortality [9]. PWB consists of autonomy, self-acceptance, purpose in life, environmental mastery, positive relationships and personal growth [10]. However, there is an evidence that negative emotions, but not the positive ones, have an influence on long-term survival [1, 11]. Also it has been argued that PWB does not have any direct effect on mortality [12, 13].

The influence of positive affect on health outcomes is still unclear [7]. Many studies established higher mortality risk for the people with depressive symptoms [14–18], emphasizing the importance of mental health problems when dealing with physical health outcomes [14, 18]. However, there is a lack of studies analysing associations between positive factors such as PWB and mortality in middle-income countries [6]. It is crucial to analyse PWB and mortality associations in countries with different economical status as PWB profile depends on geopolitical status of the country the person lives in [6, 19, 20]. Moreover, it is still unclear if PWB is an independent predictor of mortality, or it works through socio-demographic and other health factors [12, 13, 21]. It has been argued if PWB predicts all-kind mortality or is the link specific [9]. Gender influence on the association between PWB and mortality is also the matter of discussion [22, 23].

So the aim of this study was to examine, whether after confounding by possible socio-demographic and other risk factors, PWB is independently associated with reduced mortality.

## Methods

### Study sample

Data from the survey performed in the framework of the international HAPIEE (Health, Alcohol and Psychosocial Factors in Eastern Europe) study are presented [24]. A random sample of 10,940 Kaunas city (Lithuania) men and women aged 45–72 years, stratified by gender and age was selected from Lithuanian register of population. The response rate was 65%, thus 7115 respondents participated in this health survey from 2006 to 2008. We excluded 592 respondents because of incomplete information on PWB. Only participants who answered more than 75% of the questions were included. Missing values were changed with the average of row. Excluded persons did not differ from the rest of the cohort according to other analysed variables. In addition, 1402 (21.5%) participants with CVD at baseline (coronary heart disease (CHD) or stroke) were excluded from the analysis of CVD mortality risk. The final number of participants included in the analysis was 6523 for all-cause mortality, and 5121 for CVD mortality. The study was approved by the Ethics Committee at University College London, UK and by Kaunas Regional Biomedical Research Ethics Committee.

## Measures

### PWB scale

PWB was evaluated by a Control Autonomy Self-realization and Pleasure (CASP-12) questionnaire [25]. It is composed of 12 statements. Participants indicate how often (often, sometimes, not often, never) each statement applies to them. The total score ranges from 12 to 48, where a higher score represents higher PWB. The internal consistency of the scale was good (Cronbach’s *α* = 0.74). The scale was dichotomised to produce two groups of PWB, one consisting participants with high PWB and the other of participants with lower PWB. Participants were classified as having a higher PWB if the CASP-12 score was higher or equal to the median: ≥ 40 in men and ≥ 38 in women.

### Depressive symptoms

Depressive symptoms were measured using the ten-item Center for Epidemiologic Studies Depression Scale (CES-D 10) [26]. Specially trained personnel filled in questionnaires by interviewing the respondents. The subjects were asked to evaluate the presence of ten depressive symptoms during the past week on a two-point scale: yes or no. Each symptom was scored 0 (no) or 1 (yes), resulting in a total score of 0–10. The subjects with CES-D 10 scores of 4 or more were classified as having depressive symptoms [27, 28]. The scale showed good enough internal consistency (Cronbach’s *α* = 0.72).

### Other variables determined using the questionnaire

The standard questionnaire included questions regarding the respondent’s age, education, self-rated health, quality of life, smoking status, alcohol consumption, physical activity, number of children, employment status, etc. These variables were determined using the questionnaire by HAPIEE study protocol [24]. The cutoffs of variables were used in other scientific analysis too [29–31].

Education was classified into five education levels: primary, vocational, secondary, college and university. Marital status of all study participants was divided into five groups: single, married, cohabited, divorced and widowed. Participants were classified as not having children, having one, two or three and more children. Employment status was derived by classifying participants into employed, employed-retired, employed-disabled, disabled, retired and unemployed groups. Study participants were also classified by social activity and social participation. Social activity was evaluated by statements about participating in clubs, going to church, restaurants, theatres, sports clubs, etc. Participants were divided into three groups: low, moderate and high social activity. Social participation shows the percentage of the sample being a member of a social organization. An indicator of material deprivation was assessed by questions about how often the person’s household had difficulties in buying enough food or clothes and paying bills for housing, heating and electricity. A higher deprivation score means a lower level of deprivation.

Smoking habits were assessed according to the current smoking status. The respondents were classified into three groups: smokers, former smokers and never smokers. A subject who smoked at least one cigarette per day was classified as current smoker. Alcohol consumption was measured by asking participants how often they drink alcohol: every day, 2–4 times per week, once per week, 1–3 times per month, less than once per month, never. Physical activity was determined by meantime spent per week during leisure time in winter and summer for walking, moderate and hard work, gardening and other physical activities. Physical activity was determined by the mean length of time spent per week during leisure time in winter and summer for walking, moderate and hard work like gardening and other physical activities. The respondents were categorized into two groups according to their physical activity in leisure time: physically active (10 h or more) and inactive (< 10 h).

### Measurements

Blood pressure was measured three times, using an oscillometric device (Omron M5-I) after a 5 min rest. The mean of three systolic and diastolic blood pressure was used. Arterial hypertension was defined as systolic blood pressure ≥ 140 mmHg and/or diastolic blood pressure ≥ 90 mmHg, or normal blood pressure (< 140/90 mmHg) if the person had taken antihypertensive drugs within the last 2 weeks. Body Mass Index (BMI) was calculated as weight (kg) divided by the square meters of height.

### Laboratory analyses

Biochemical analyses were conducted for > 6 h fasting participants. Concentration of glucose in capillary blood was determined by an individual glucometer “Glucotrend” [32]. Serum samples were analysed centrally in one batch in the WHO Regional Lipid Reference Centre, Institute of Clinical and Experimental Medicine, Prague (Czech Republic). Lipid concentrations [low-density lipoprotein (LDL) cholesterol, high-density lipoprotein (HDL) cholesterol, and triglycerides] in serum were measured, using a conventional enzymatic method.

### Cardiovascular diseases

Coronary heart disease (CHD) at baseline was determined by: (1) a documented history of myocardial infarction (MI) and/or ischemic changes on electrocardiogram (ECG) coded by Minnesota codes (MC) 1–1 or 1–2 [33]; (2) angina pectoris as defined by G. Rose’s questionnaire (without MI and/or MC 1–1 or 1–2) [34]; (3) ECG findings coded by MC 1–3, 4–1, 4–2, 4–3, 5–1, 5–2, 5–3, 6–1, 6–2, 7–1, or 8–3 (without MI and/or MC 1–1, 1–2 and without angina pectoris). Previous stroke was determined according to a documented history of stroke.

### Follow-up

The data from the regional mortality register based on death certificates were used for the follow-up of participants. Only underlying causes of death were considered. Death certificates based on medical documentation were sufficiently valid and complete [35]. Deaths between the baseline survey date and December 31st 2015 were registered. Causes of death were coded by the International Classification of Diseases (ICD) (versions 9 and 10): deaths of CVD included codes 390–458 of ICD-9 and codes I00–I99 of ICD-10. Over the period of 2006–2015, 747 death cases from any cause (484 men and 263 women) and 163 deaths from CVD (excluding those with previous CVD at entry) (114 men and 49 women) were registered. The mean duration of follow-up was 8.16 ± 1.6 years (7.93 ± 1.9 years among men and 8.35 ± 1.3 years among women).

### Statistical analysis

Descriptive statistics were calculated for variables included in the data analysis. The prevalence of lifestyle factors was compared in gender and PWB groups via *χ*^2^ tests. Mean differences were tested via *t*-test.

The Kaplan–Meier survival curves for cumulative all-cause mortality and CVD mortality according to the categories of PWB for men and women were plotted. Log rank test was performed to evaluate difference between PWB categories.

Hazard ratios (HR) and 95% confidence intervals (CI) were estimated by the multivariate Cox proportional hazards regression for all-cause and CVD mortality separately for men and women. PWB dichotomization by median score was chosen because it resulted in increased power and estimates of the HR and the cutpoint were less biased when compared with the univariate approach.

For multivariate analysis, we entered all variables that were significantly associated with all-cause and CVD mortality in the univariate Cox proportional hazards regression analysis. Attained age in months was used as time-scale. Several models were assessed. For all-cause mortality: Model 1 for men and women-adjusted for age. Model 2 adjusted for all the variables in Model 1 plus socio-demographic and socio-economic factors: for men—marital status, education, employment status, social activity, material deprivation, number of children, and social participation; for women—education, employment status, social activity, and material deprivation. Model 3 adjusted for all the variables in Model 2 plus lifestyle and biologic factors: for men—HDL cholesterol, LDL cholesterol, glucose, triglycerides, smoking habits, alcohol consumption, physical activity, depression symptoms, and arterial hypertension; for women—BMI, HDL cholesterol, LDL cholesterol, glucose, triglycerides, smoking habits, physical activity, and arterial hypertension. For cardiovascular mortality: Model 1 for men and women—adjusted for age. Model 2 adjusted for all the variables in Model 1 plus socio-demographic and socio-economic factors: for men—marital status, education, employment status, social activity; for women—employment status, social activity. Model 3 adjusted for all the variables in Model 2 plus lifestyle and biologic factors: for men—triglycerides, smoking habits, physical activity and arterial hypertension; for women—BMI, HDL cholesterol, LDL cholesterol, glucose, and systolic blood pressure.

The relative impact for each covariate on the association between PWB and all-cause mortality was assessed using the formula as described by Lynch et al. [36] (HR_modelA_ − HR_modelB_)/(HR_modelA_ − 1), where model A is the unadjusted model and where model B includes the covariate.

Data were analysed using the IBM SPSS Statistics 20.0. *p* < 0.05 values were considered statistically significant.

## Results

Table 1 presents the baseline characteristics of the study participants by PWB groups (lower PWB and higher PWB). Significant differences in biological CVD risk factors were ascertained according to the level of PWB and in gender groups. Moreover, associations were found in the distribution of the socio-demographic, and socio-economic factors across the two categories of PWB. Lifestyle habits also varied across the two categories of PWB and gender groups (Table 1).

**Table 1:** Baseline characteristics of the study population by the level of psychological well-being (PWB)

	Men		Women	
	Lower PWB	Higher PWB	Lower PWB	Higher PWB
	< Median	≥ Median	< Median	≥ Median
Mean age [years (SD)]	60.8 (7.6)	60.4 (7.6)	61.1 (7.5)	59.8 (7.6)***
BMI, kg/m^2^ [mean (SD)]	28.5 (4.9)	28.5 (4.4)	30.4 (5.9)	29.7 (5.6)***
Triglycerides [mmol/l (SD)]	1.51 (1.02)	1.44 (0.92)*	1.50 (0.82)	1.41 (0.79)**
HDL cholesterol [mmol/l (SD)]	1.40 (0.39)	1.40 (0.36)	1.55 (0.38)	1.58 (0.37)**
LDL cholesterol [mmol/l (SD)]	3.68 (1.03)	3.71 (0.97)	3.88 (1.05)	3.90 (1.04)
Glucose [mmol/l (SD)]	5.85 (1.37)	5.80 (1.21)	5.89 (1.29)	5.81 (1.13)
Arterial hypertension (%)	74.9	73.2	67.0	60.5***
Smoking status (%)				
Current	33.9	26.9*	10.1	9.7
Former	30.3	32.0	6.9	7.2
Never	35.8	41.0*	83.0	83.1
Alcohol consumption (%)				
Every day	7.5	5.2*	0.8	1.2
2–4 times per week	14.6	17.7*	2.1	3.3*
Once a week	15.7	16.6	5.8	8.1
1–3 times per month	33.0	33.7	27.1	34.3*
Less than once a month	23.3	21.8	53.9	48.0*
Never	5.9	5.0	10.3	5.1*
Physically active (%)	64.5	72.4***	76.5	84.2
Marital status (%)				
Married	82.0	85.6*	53.3	57.8*
Single	2.4	1.4	5.9	5.6
Cohabiting	1.4	1.4	1.1	0.6
Divorced	8.1	7.0	17.0	15.1
Widowed	6.1	4.6	22.7	20.9
Number of children (%)				
0	7.7	4.8*	11.2	10.2
1	26.4	23.1*	31.4	27.1*
2	54.1	58.1*	47.7	53.8*
3 and more	11.8	14.0	9.7	8.9
Education level (%)				
Primary	7.6	6.8	9.6	5.1*
Vocational	11.2	9.0*	9.9	6.7*
Secondary	32.6	29.2*	28.5	22.4*
College	18.0	18.0	25.7	28.5
University	30.6	37.0*	26.3	37.3*
Employment status (%)				
Employed	35.6	45.3*	26.7	37.2*
Employed-retired	15.7	23.3*	11.2	17.1*
Employed-disabled	5.1	3.3*	3.6	2.9
Unemployed	3.6	2.2*	3.6	3.5
Retired	25.3	20.3*	40.7	32.4*
Disabled	14.7	5.6*	14.2	6.9*
Social participation (%)	85.6	81.6**	9.8	15.6***
Social activity (%)				
Low	42.1	26.7*	38.1	20.0*
Moderate	34.7	32.9	40.6	38.9
High	23.2	40.4*	21.3	41.1*
Material deprivation (%)	33.5	13.9***	49.7	26.8***
Depressive symptoms (%)	26.7	5.1***	49.6	13.7***
CHD (%)	25.3	16.1***	27.9	17.8***

*CHD* coronary heart disease, *SD* standard deviation, BMI Body Mass Index, HDL high-density lipoprotein, LDL low-density lipoprotein

*p < 0.05, **p < 0.01, ***p < 0.001 as compared to the group with lower psychological well-being (distributions were compared using *χ*^2^, mean values were compared using *t-*test)

**Fig. 1:**
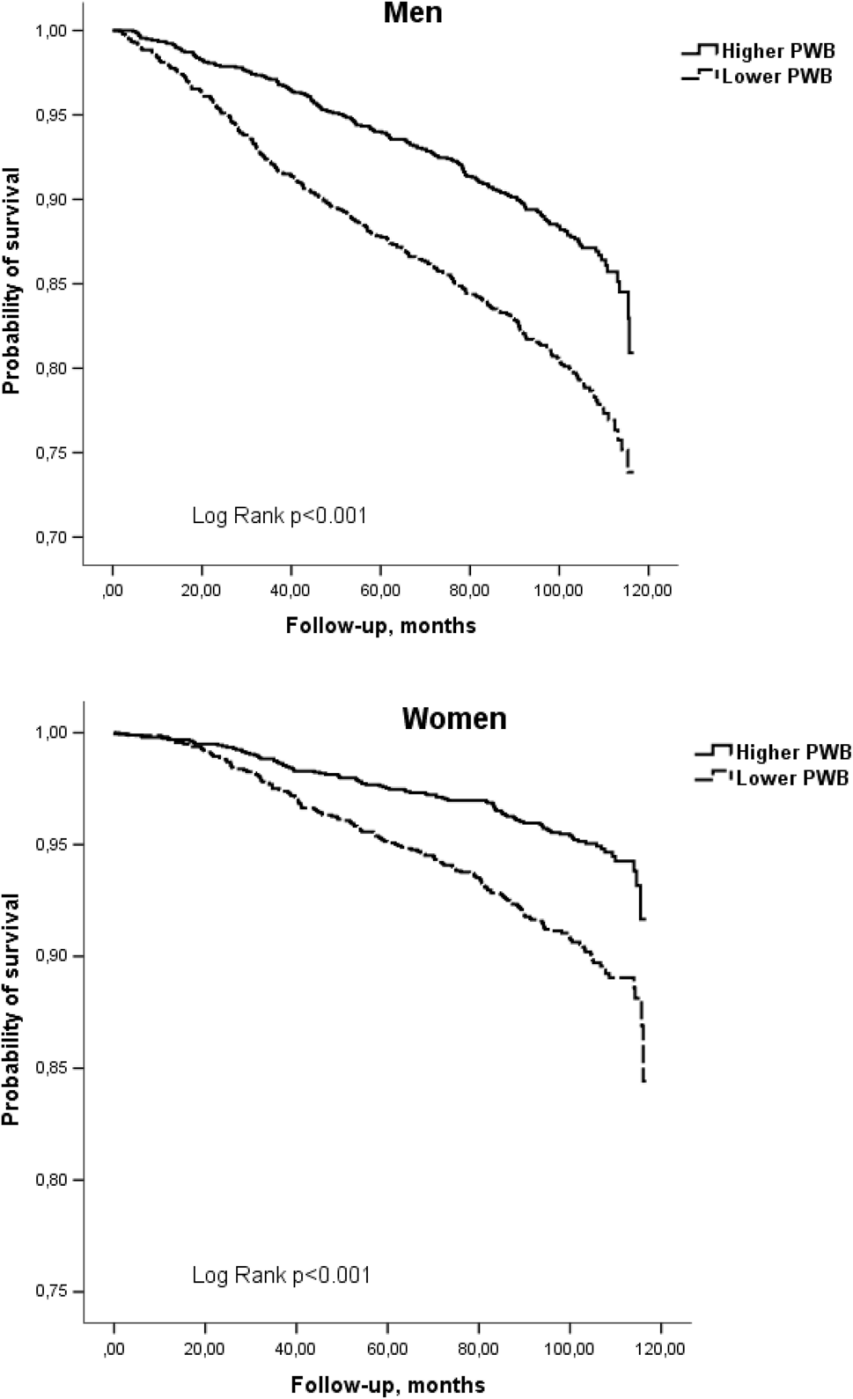
Kaplan–Meier survival (all-cause mortality) curves according to the categories of psychological well-being for men and women, adjusted for age

**Fig. 2:**
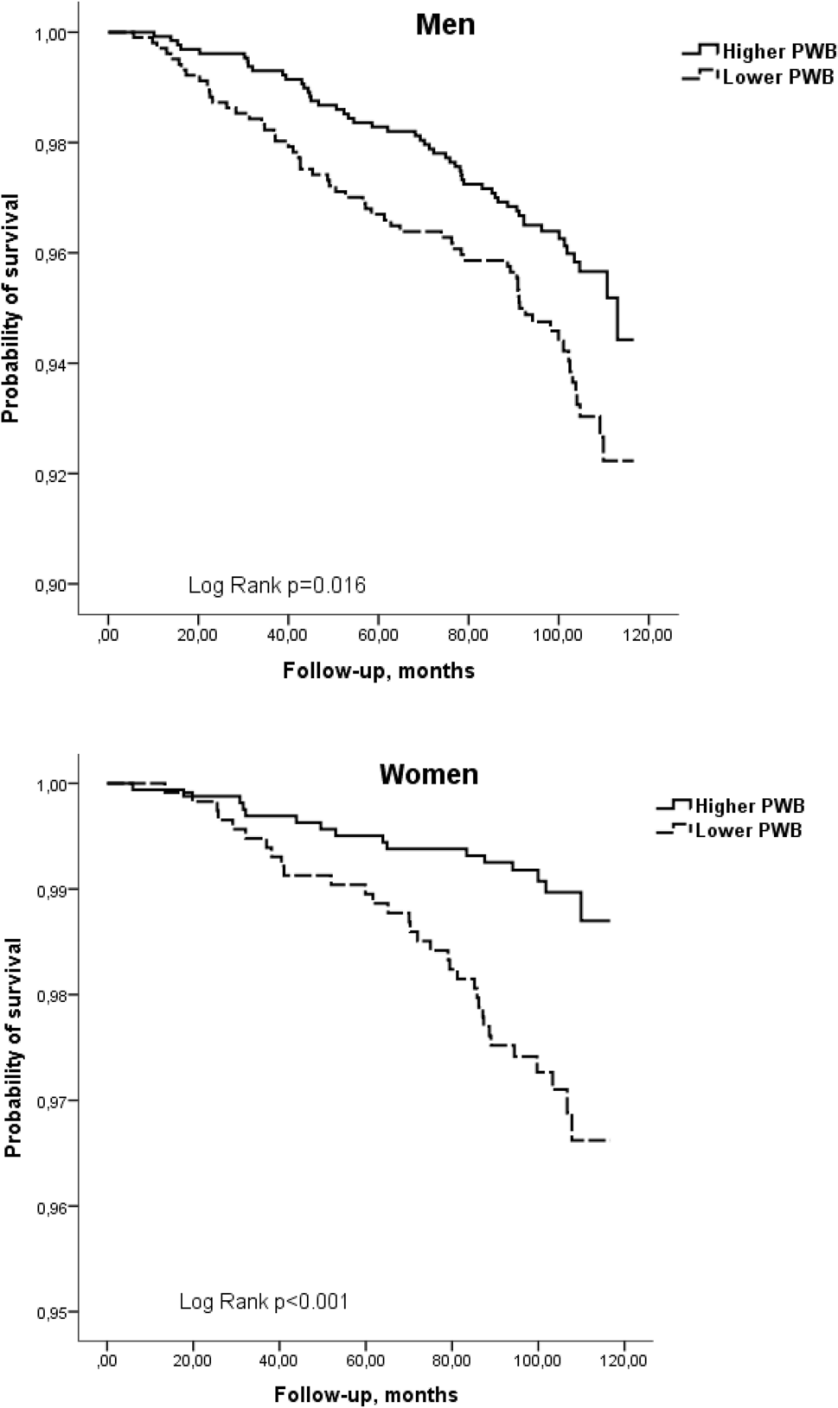
Kaplan–Meier survival (cardiovascular mortality) curves according to the categories of psychological well-being for men and women, adjusted for age

The Kaplan–Meier survival curves for cumulative all-cause mortality and mortality from CVD according to the categories of PWB for men and women, adjusted for age are presented in Figs. 1 and 2. Log-ranked test revealed that the cumulative survival rate within 8.16 years of follow-up differed significantly (*p* < 0.016) for the two PWB groups that represent successive levels of higher PWB, with lower PWB predicting shorter survival.

Table 2 presents multivariate association of PWB with all-cause and cardiovascular mortality in men and women, after adjustment for socio-demographic, socio-economic factors and lifestyle and biologic risk factors. Men and women with higher PWB had the lowest age-adjusted risk of all-cause mortality (respectively, HR 0.58 and HR 0.56) and risk of CVD mortality (respectively, HR 0.63 and HR 0.41), compared to respondents with lower PWB (Model 1). After adjustment by age plus socio-economic factors and lifestyle factors (Model 2) the women with higher PWB had the lowest age-adjusted risk of all-cause mortality (HR 0.69) and risk of CVD mortality (HR 0.51), compared to women with lower PWB. Additional adjustment by lifestyle and biologic risk factors (Model 3) attenuated the risk in women group, but the pattern remained. However, in men after additional adjustment, significant differences of higher and lowest PWG groups were found in all-cause mortality, but not in mortality from CVD. So, a clear effect of higher PWB to all-cause mortality was ascertained in men and women, yet for CVD mortality there was a clear effect only in women group.

**Table 2:** Multivariate association of psychological well-being with cardiovascular and all-cause mortality in men and women, after adjustment for socio-demographic, socio-economic, lifestyle and biologic risk factors

Psychological well-being (PWB)	No. of deaths (*n*)	Model 1	HR (95% CI)	Model 2	HR (95% CI)	Model 3	HR (95% CI)
		Men	Women	Men	Women	Men	Women
				All-cause	Mortality		
Lower PWB	Men 289 (1388)	1.0 (ref.)	1.0 (ref.)	1.0 (ref.)	1.0 (ref.)	1.0 (ref.)	1.0 (ref.)
	Women 162 (1604)						
Higher PWB	Men 195 (1552)	**0.58**	**0.56**	**0.70**	**0.69**	**0.77**	**0.73**
	Women 101 (1979)	**(0.49–0.70)**	**(0.44–0.72)**	**(0.57–0.85)**	**(0.53–0.91)**	**(0.62–0.94)**	**(0.56–0.96)**

Psychological well-being (PWB)	No. of deaths (n)	Model 1	HR (95% CI)	Model 2	HR (95% CI)	Model 3	HR (95% CI)
		Men	Women	Men	Women	Men	Women
				Cardiovascular	Mortality^a^		
Lower PWB	Men 62 (1037)	1.0 (ref.)	1.0 (ref.)	1.0 (ref.)	1.0 (ref.)	1.0 (ref.)	1.0 (ref.)
	Women 33 (1157)						
Higher PWB	Men 52 (1302)	**0.63**	**0.41**	0.88	**0.51**	0.98	**0.53**
	Women 16 (1625)	**(0.43–0.91)**	**(0.22–0.74)**	(0.67–1.16)	**(0.32–0.79)**	(0.72–1.33)	**(0.33–0.87)**

Model 1 adjusted for age

Model 2 adjusted for all the variables in Model 1 plus socio-demographic and socio-economic factors

Model 3 adjusted for all the variables in Model 2 plus lifestyle and biologic factors. For details see in “Methods” and “Statistical analysis”

Bold typeface indicates significance

*CI* confidence interval, *HR* hazard ratio, *CI* confidence interval

^a^Without CVD at baseline survey

Table 3 shows relative impact of covariates on association between PWB and all-cause mortality in men and women. In men’s group, the most notable relative impact on the association was found for social activity (12% explained variance), depressive symptoms (9%), physical activity (9%), employment status (7%). In women’s group, the most notable relative impact on the association was found for social activity (15%) and employment status (13%). As for the other covariates, the current analysis revealed that mediating effects on the association between PWB and mortality were less significant.

**Table 3:** Relative impact of covariates on association between psychological well-being and all-cause mortality in men and women

Covariate	Men			Women		
	HR	95% CI	Relative impact on association (%)	HR	95% CI	Relative impact on association (%)
No covariate^a^	0.58	0.49–0.7		0.56	0.44–0.72	
Marital status	0.59	0.49–0.70	1			
Education	0.60	0.5–0.72	4	0.58	0.45–0.74	5
Number of children	0.59	0.49–0.71	1			
Employment status	0.61	0.55–0.8	7	0.62	0.48–0.79	13
Material deprivation	0.60	0.49–0.72	3	0.59	0.46–0.76	7
Social participation	0.59	0.49–0.71	1			
Social activity	0.63	0.53–0.76	12	0.62	0.4–0.81	15
Body Mass Index				0.57	0.44–0.73	2
HDL cholesterol	0.61	0.5–0.73	6	0.58	0.45–0.74	4
LDL cholesterol	0.60	0.5–0.72	4	0.57	0.44–0.73	1
Glucose	0.58	0.49–0.7	0	0.56	0.43–0.72	0
Triglycerides	0.59	0.49–0.7	1	0.56	0.43–0.72	0
Arterial hypertension	0.58	0.49–0.7	0	0.57	0.45–0.74	3
Alcohol consumption	0.59	0.49–0.71	2			
Smoking habits	0.61	0.51–0.73	6	0.56	0.44–0.72	0
Physical activity	0.62	0.52–0.74	9	0.58	0.45–0.74	4
Depressive symptoms	0.62	0.51–0.75	9			

*CI* confidence interval, *HR* hazard ratio, *HDL* high-density lipoproteins, *LDL* low-density lipoproteins

^a^Adjusted by age

## Discussion

PWB was associated with reduced risk of all-cause mortality and CVD mortality in Lithuanian adults who were CVD-free at baseline. Survival curves revealed PWB to be a protective factor against mortality during more than 8 years. The link was ascertained in previous studies too [5–8]. The association between PWB and all-cause mortality held even after adjusting for potential socio-demographic, socio-economic, biological and behavioural covariates. So the study results argue that PWB is associated with better survival regardless of negative affect, unlike some previous studies [11, 37].

The study raised some more interesting questions. First, gender issue in the association between PWB and CVD mortality. In this study after confounding for possible covariates, PWB was associated with mortality from CVD in women, but not in men. The link lost its significance after adjusting for socio-demographic, socio-economic, biological and behavioural factors. Previous study revealed that Ikigai, known as well-being in the culture of Japan, effect was stronger for men than women [22]. But we did not manage to find a study showing analogic to our results. Also it could not be pointed to small number of death cases, because the deaths from CVD number in women group were even smaller, compared to men. So the reason might be different correlates of PWB connected to mortality. As previous studies found, sex differences in association between PWB and CVD, might be contributed by different biological risk factors in genders [38].

Possible confounders were analysed in this study too. Some different covariates were established according to gender. However highest relative impact on association was ascertained for social activity both in men and women. It is known that social isolation has a direct effect on mortality [39]. In men, depressive symptoms and smoking habits were important covariates in association between PWB and mortality, but it was not significant in women. In association of PWB and all-cause mortality, employment status was an important covariate in both genders. Employment status in this study covers disability aspect as one of the categories is disabled. Previous studies established that disability and mobility impairment is an important associate of mortality [4], so it is seen in results of this study too. Moreover, employment status was more significant in women compared to men, maybe because we had higher rates of unemployment and disability between women compared to men, as in previous studies [40].

After more than 8 years of follow-up the conclusion can be drawn about independent association between PWB and survival. As was said before, a protective role of high positive PWB should be taken into account [6]. Although mostly important correlates were controlled, it is an observational study and causal relationships cannot be generated.

The present study has several strengths. It is a longitudinal study of middle-aged and older Lithuanians analysing association between PWB and mortality with quite long follow-up—more than 8 years. For our knowledge this is the first attempt in Baltic countries to analyse the link between PWB and longevity. Also it examined PWB associations with longevity independent from negative affect, adjusting for depressive symptoms. Not only socio-demographic, socio-economic and behavioural factors were included into analysis, but many classical biological risk factors for CVD mortality too. Moreover, this study is sensitive to gender and proved some gender differences in association between PWB and mortality.

This study has several limitations too. The study included representative sample of Kaunas city; however, it does not represent the national sample and cannot be generalized to any other country. Besides, not all participants filled the PWB scale and were excluded from the analysis. Yet, those participants did not differ from other participants significantly. Finally, the response rates lower than 70% might have resulted in selection bias and potential underestimation of the prevalence of worse psychological well-being and CVD, if non-response was associated with a more adverse risk profile. PWB was measured only in the beginning of the study and the possible fluctuation was not controlled [4]. There might be some more potential confounding factors which were not included in to study, such as predictors of PWB—personality [41] or stress [42].

## Conclusion

PWB is an important predictor of longevity controlling for well-recognized risk factors such as age, education, CVD, social status, marital status, and lifestyle. Positive PWB influence on mortality is especially important in older population. Not only depressive symptoms, but also positive psychological factors should be taken into account when screening older people to prevent negative health outcomes.

## References

[1] Deary IJ, Weiss A, Batty GD (2010). Intelligence and personality as predictors of illness and death: how researchers in differential psychology and chronic disease epidemiology are collaborating to understand and address health inequalities. Psychol Sci Public Interest.

[2] Karraker A, Schoeni RF, Cornman JC (2015). Psychological and cognitive determinants of mortality: evidence from a nationally representative sample followed over thirty-five years. Soc Sci Med.

[3] Consedine NS, Moskowitz JT (2007). The role of discrete emotions in health outcomes: a critical review. Appl Prev Psychol.

[4] Zaninotto P, Wardle J, Steptoe A (2016). Sustained enjoyment of life and mortality at older ages: analysis of the English Longitudinal Study of Ageing. BMJ.

[5] Chida Y, Steptoe A (2008). Positive psychological wellbeing and mortality: a quantitative review of prospective observational studies. Psychosom Med.

[6] Steptoe A, Deaton A, Stone AA (2015). Psychological wellbeing, health, ageing. Lancet.

[7] Gana K, Broc G, Saada Y, Amieva H, Quintard B (2016). Subjective wellbeing and longevity: findings from 22-year cohort study. Psychosom Res.

[8] Sadler ME, Miller CJ, Christensen K, McGue M (2011). Subjective wellbeing and longevity: a co-twin control study. Twin Res Hum Genet.

[9] Kimm H, Sull JW, Gombojav B, Yi SW, Ohrr H (2012). Life satisfaction and mortality in elderly people: the Kangwha Cohort Study. BMC Public Health.

[10] Ryff CD, Singer BH (2008). Know thyself and become what you are: a eudaimonic approach to psychological well-being. J Happiness Stud.

[11] Ortega FB, Lee DC, Sui X, Kubzansky LD (2010). Psychological wellbeing, cardiorespiratory fitness, and long-term survival. Am J Prev Med.

[12] Liu B, Floud S, Pirie K, Green J, Peto R, Beral V, Million Women Study Collaborators (2016). Does happiness itself affect mortality? The prospective UK million women study. Lancet.

[13] Koopmans TA, Geleijnse JM, Zitman FG, Giltay EJ (2010). Effects of happiness on all-cause mortality during 15 years of follow-up: the Arnhem elderly study. J Happiness Stud.

[14] Jia H, Lubetkin EI (2017). Incremental decreases in quality-adjusted life years (QALY) associated with higher levels of depressive symptoms for US adults aged 65 years and older. Health Qual Life Outcomes.

[15] Murphy RA, Hagaman AK, Reinders I (2016). Depressive trajectories and risk of disability and mortality in older adults: longitudinal findings from the health, aging, and body composition study. J Gerontol Med Sci.

[16] Huffman JC, Celano CM, Beach SR, Motiwala SR, Januzzi JL (2013). Depression and cardiac disease: epidemiology, mechanisms, and diagnosis. Cardiovasc Psychiatry Neurol.

[17] McDermott MM, Guralnik JM, Tian L (2016). Incidence and prognostic significance of depressive symptoms in peripheral artery disease. J Am Heart Assoc.

[18] White J, Zaninotto P, Walters K (2016). Duration of depressive symptoms and mortality risk: the English Longitudinal Study of Ageing (ELSA). Br J Psychiatry.

[19] Schönfeld P, Brailovskaia J, Margraf J (2017). Positive and negative mental health across the lifespan: a cross-cultural comparison. Int J Clin Health Psychol.

[20] Karasawa M, Curhan K, Markus H, Kitayama S, Love G, Radler B, Ryff C (2011). Cultural perspectives on aging and well-being: a comparison of Japan and the United States. Int J Aging Hum Dev.

[21] Diener E, Chan MY (2010). Happy people live longer: subjective wellbeing contributes to health and longevity. Appl Psychol Health Wellbeing.

[22] Tanno K, Sakata K, Ohsawa M (2009). Associations of ikigai as a positive psychological factor with all-cause mortality and cause-specific mortality among middle-aged and elderly Japanese people: findings from the Japan Collaborative Study. J Psychosom Res.

[23] Sapranaviciute-Zabazlajeva L, Tamosiunas A, Luksiene D, Virviciute D (2016). The links between cardiovascular risk and psychological wellbeing in elderly. Int J Med Health Biomed Bioeng Pharm Eng.

[24] Peasey A, Bobak M, Kubinova R (2006). Determinants of cardiovascular disease and other non-communicable diseases in Central and Eastern Europe: rationale and design of the HAPIEE study. BMC Public Health.

[25] Kim GR, Netuveli G, Blane D, Peasey A, Malyutina S, Simonova G (2015). Psychometric properties and confirmatory factor analysis of the CASP-19, a measure of quality of life in early old age: the HAPIEE study. Aging Ment Health.

[26] Carpenter JS, Andrykowski MA, Wilson J (1998). Psychometrics for two short forms of the center for epidemiologic studies: depression scale. Issues Mentl Health Nurs.

[27] Sapranaviciute-Zabazlajeva L, Reklaitiene R, Tamosiunas A, Baceviciene M, Virviciute D, Peasey A (2014). Correlates of depressive symptoms in urban middle-aged and elderly Lithuanians. Soc Psychiatry Psychiatr Epidemiol.

[28] Irwin M, Artin KH, Oxman MN (1999). Screening for depression in the older adult: criterion validity of the 10-item Center for Epidemiological Studies Depression Scale.(CES-D). Arch Intern Med.

[29] Sapranaviciute-Zabazlajeva L, Luksiene D, Virviciute D, Bobak M, Tamosiunas A (2017). Link between healthy lifestyle and psychological well-being in Lithuanian adults aged 45–72: a cross-sectional study. BMJ Open.

[30] Luksiene D, Tamosiunas A, Virviciute D, Bernotiene G, Peasey A (2015). Anthropometric trends and the risk of cardiovascular disease mortality in a Lithuanian urban population aged 45–64 years. Scand J Public Health.

[31] Tamosiunas A, Baceviciene M, Reklaitiene R, Radisauskas R, Jureniene K, Azaraviciene A, Luksiene D, Malinauskiene V, Daugeliene E, Sapranaviciute-Zabazlajeva L (2012). Cardiovascular risk factors and cognitive function in middle aged and elderly Lithuanian urban population: results from the HAPIEE study. BMC Neurol.

[32] Norkus A, Ostrauskas R, Sulcaite R, Baranauskiene E, Baliutaviciene D (2000). Classification and diagnostics of diabetes mellitus (methodology recommendations). Lith Endocrinol.

[33] Prineas RJ, Crow RS, Blackburn H (1982). The Minnesota code. Manual of electrocardiographic findings.

[34] Rose GA, Blackburn H, Gillum RF, Prineas RJ (1982). Cardiovascular survey methods.

[35] Radisauskas R, Prochorskas R, Grabauskas V, Bernotiene G, Tamosiunas A, Veryga A (2011). Recent heavy alcohol consumption at death certified as ischemic heart disease: correcting mortality data from Kaunas (Lithuania). Alcohol Alcohol.

[36] Lynch JW, Kaplan GA, Cohen RD, Tuomilehto J, Salonen JT (1996). Do cardiovascular risk factors explain the relation between socioeconomic status, risk of all-cause mortality, cardiovascular mortality, and acute myocardial infarction?. Am J Epidemiol.

[37] Brummett BH, Boyle SH, Siegler IC, Williams RB, Mark DB, Barefoot JC (2005). Ratings of positive and depressive emotion as predictors of mortality in coronary patients. Int J Cardiol.

[38] Steptoe A, Demakakos P, de Oliveira C, Wardle J (2012). Distinctive biological correlates of positive psychological well-being in older men and women. Psychosom Med.

[39] Holt-Lunstad J, Smith TB, Baker M, Harris T, Stephenson D (2015). Loneliness and social isolation as risk factors for mortality: a meta analytic review. Perspect Pychol Sci.

[40] Pinquart M, Sörensen S (2001). Gender differences in self-concept and psychological wellbeing in old-age: a meta-analysis. J Gerontol B Psychol Sci Soc Sci.

[41] Schönfeld P, Brailovskaia J, Bieda A, Zhang XC, Margraf J (2016). The effects of daily stress on positive and negative mental health: mediation through self-efficacy. Int J Clin Health Psychol.

[42] Huppert F (2009). Psychological well-being: evidence regarding its causes and consequences. Appl Psychol Health Well-Being.

